# Two-Year Longitudinal Motor Performance of Very Preterm and/or Very-Low-Birth-Weight Infants in Suriname

**DOI:** 10.3390/children12040414

**Published:** 2025-03-26

**Authors:** Anjo J. W. M. Janssen, Maria J. A. J. Fleurkens-Peeters, Reinier P. Akkermans, Se-Sergio M. Baldew, Maria W. G. Nijhuis-van der Sanden, Wilco C. W. R. Zijlmans

**Affiliations:** 1 Pediatric Physical Therapy, Department of Rehabilitation, Amalia Children’s Hospital, Radboud University Medical Center, 6525 GA Nijmegen, The Netherlands; ria.nijhuis-vandersanden@radboudumc.nl; 2 Pediatric Physical Therapy, Department of Rehabilitation, Academic Hospital Paramaribo, Flustraat 1, Paramaribo, Suriname; 3 IQ Health Science Department, Radboud University Medical Center, 6525 EP Nijmegen, The Netherlands; reinier.akkermans@radboudumc.nl; 4 Research Institute for Medical Innovation, Department of Primary and Community Care, Radboud University Medical Center, 6525 EZ Nijmegen, The Netherlands; 5 Physical Therapy Department, Faculty of Medical Sciences, Anton de Kom University of Suriname, Prof. W.J. Kernkampweg 5, Paramaribo, Suriname; sergio.baldew@uvs.edu; 6 Discipline of Pediatrics, Faculty of Medical Sciences, Anton de Kom University of Suriname, Prof. W.J. Kernkampweg 5, Paramaribo, Suriname; wilco.zijlmans@uvs.edu

**Keywords:** premature birth, follow-up, motor, low- or middle-income country, Bayley Scales of Infant and Toddler Development, longitudinal, cohort, regression analyses

## Abstract

**Background/Objectives:** Follow-up studies in very preterm infants are common, but fewer studies are situated in low- or middle-income countries. In a prospective cohort study, we explored longitudinal motor performance trajectories and influencing factors, including an early motor intervention program. Very preterm infants (gestational age < 32 weeks and/or very-low-birth-weight < 1500 g) in the middle-income country of Suriname were included. **Methods:** We assessed 149 (49.7% boys) infants (mean gestational age 29^+6^, mean birth weight 1271 g) at 3, 12, and 24 months with the Bayley Scales of Infant and Toddler Development for fine motor (FM), gross motor (GM), and composite scores (CSs). Influencing perinatal and environmental factors were explored. Delayed-scoring infants were referred to a motor intervention program. Data were analyzed using mixed-model linear regression. **Results:** The Bayley mean FM and GM scores decreased between 3 and 12 months and stabilized at 24 months. The mean CS at 3, 12, and 24 months was 102.3, 92.7, and 92.2, respectively. The latter two were significantly below the reference values (100, SD 15, *p* < 0.01). Birth weight z-scores significantly influenced FM (*p* = 0.013) and CS (*p* = 0.009); a lower birth weight was associated with initially lower scores and a smaller decline over time than a higher birth weight. The motor intervention program (*n* = 54) showed no significant interaction effects at all time points after correction for frequency of interventions (no; 1–5; >5 interventions). **Conclusions:** Motor performance was normal at 3 months and delayed at 12 and 24 months. Birth weight, but not the early intervention program, influenced longitudinal motor trajectories. We recommend follow-up of motor performance and suggest adding the Prechtl General Movement assessment at 3 months of age. The clinical implementation of the early motor invention program needs additional studies to reach an adequate training level.

## 1. Introduction

Motor performance can be assessed with a motor test. In a validated motor test, the individual score is compared to a reference group of typically developing children and is often classified as normal or delayed. Delayed motor performance, in very preterm infants (VPT: <32 weeks) and/or in very-low-birth-weight (VLBW: <1500 g) infants is common and has been reported in two systematic reviews [1,2]. Most studies were situated in high-income countries (HICs) in North America and Europe; others were conducted primarily in Asia and Brazil [3,4]. Fewer studies were situated in low- or middle-income countries (LMICs) such as Uganda and Malawi [5,6].

Differences in multiple economic and socio-cultural factors between LMICs and HICs may impact the motor performance outcomes of VPT and/or VLBW infants [3,7]. First, neonatal care facilities in LMICs are often limited, leading to increased mortality and morbidity rates [8]. For example, technical possibilities such as the minimally invasive surfactant procedure and hypothermia treatment are either not available or implementation is delayed. Second, the gestational age (GA) at which active intervention after delivery is decided is between 23 and 24 weeks in HICs, but in LMICs, a GA of 26 to 27 weeks is advised [9]. This discrepancy might lead to differences in outcomes since infants born with a lower GA are more prone to morbidity [2,10]. In addition, cultural differences in child-rearing practices, such as sleeping time and parental beliefs regarding the outcome of their infant born preterm, might differ [11,12,13,14]. As such, data on motor performance outcome studies in HICs are not directly comparable to data from similar studies conducted in LMICs. It seems necessary that more follow-up studies of VPT and/or VLBW in LMICs are conducted. We hypothesized that Surinamese VPT and/or VLBW infants would also have delayed motor performance and could benefit when referred for an early intervention program by a pediatric physical therapist.

Suriname is an upper-middle-income (UMC) Caribbean country on the South American continent, with a population of 634,431 people in 2024 and more than 10,000 infants born every year [15]. Differences in standards of medical health care are reflected in the median ages of the population. For example, in a HIC in the United States, the median age is 38 years, while in Suriname, it is 30 (2024), indicating that poor living conditions can increase biological vulnerability [16]. In Suriname, most infants (86%) are born in one of five hospitals, one of which is an academic hospital. Uncomplicated childbirth in the hospitals usually occurs with the help of a midwife. Of the infants, 14% are born preterm (<37 weeks), and 15% are born with a low birth weight (<2500 g) [17]. About 3.9% are born VPT and/or with a VLBW. Newborns are generally actively treated in the neonatal intensive care unit (NICU) if the birth weight (BW) is ≥750 g and/or GA ≥ 27 weeks.

Follow-up of motor performance in preterm infants is important because there is high variability in the longitudinal motor performance scores of individual infants [18]. Longitudinal follow-up with repeated assessments is necessary to make appropriate decisions regarding motor interventions. Intervention programs have shown improvements in motor performance [19,20,21,22]. In 2012, a follow-up program was introduced in Suriname. Motor performance was assessed in preterm infants using a valid test, and delayed-scoring infants were referred for an early motor intervention program (EIP). In this program, parents were instructed by a pediatric physical therapist (PPT) how to stimulate motor performance while handling and playing with their infant.

This study aimed to explore the first results on longitudinal motor performance trajectories for VPT and/or VLBW infants in Suriname. Infants were assessed in a standardized follow-up program for motor performance, and infants with a delayed motor performance were referred for an EIP. The first analysis focused on the longitudinal motor performance on the Bayley Scales of Infant and Toddler Development, third version (BSID-III) fine motor scale (FM), gross motor scale (GM) and motor composite score (CS). We also wanted to explore which infant and mother characteristics, and perinatal or environmental factors, influenced the motor performance trajectories. And, finally, we explored the influence of the EIP on the motor performance trajectory in the subgroup of infants who were referred for EIP.

## 2. Materials and Methods

### 2.1. Design and Participants

A prospective longitudinal cohort study was conducted. Infants born in four hospitals in Paramaribo, the capital of Suriname, between 1 January 2014 and 31 December 2015, were included. Due to long travelling distances, the hospital in the Nickerie district was excluded. All surviving infants born VPT, GA < 32 weeks and/or with a VLBW, BW < 1500 g were eligible. Their neonatologists followed the infants after discharge. At discharge, or at the first visit, the neonatologist invited the parents to participate in the follow-up program for motor performance outcome. Parents who agreed received an invitation for the motor assessments in the pediatric department of the Rehabilitation Centre of the Academic Hospital Paramaribo.

### 2.2. Outcome Measures

#### 2.2.1. Assessment of Motor Performance

The BSID-III, FM, GM, and CS were recorded at the corrected ages (CA) of 3 months (*t*_0_), 12 months (*t*_1_), and 24 months (*t*_2_) [23]. All infants were assessed by the same bachelor-educated physical therapist, who subsequently completed a 3-year pediatric physical therapy education at master’s level, in the Netherlands (MF-P). She had more than 30 years of clinical experience with children and was trained in the assessment of the BSID-III motor scale.

The BSID-III motor scale is frequently used to investigate motor performance in preterm infants. The motor scale shows sufficient validity in different cultures and can be used for research purposes [24]. The Dutch version was recently validated for children in Suriname, and no adjustments in motor items were recommended, while the motor items were identical between the Dutch and the original US version [25]. Although the official language in Suriname, a former colony of the Netherlands, is Dutch, we used the BSID-III original version, including the US reference group. This reference group more closely resembled the diverse ethnic Surinamese population than the Dutch reference group. We have published a study showing that the US reference values are suitable in Suriname, due to lack of Surinamese reference values [26]. For the FM and GM scales, raw scores were converted into scaled scores (SSs) (range 1–19, M = 10, SD = 3). The CS (range 46–154, M = 100, SD 15) was derived from the sum of scaled FM and GM scores. The CSs were categorized as very superior (CS ≥ 130), superior (CS 120–129), high average (CS 110–119), average (CS 90–109), low average (80–89), borderline (70–79) and extremely low (≤69) scores.

#### 2.2.2. Perinatal and Environmental Factors

The referring neonatologist provided information about gender, GA, dichotomized Apgar score (AS), cutoff < 7 at 5 min postpartum, multiple birth, ventilation, defined as continuous positive pressure support or intermittent positive pressure ventilation, and clinical sepsis. To evaluate the effects of being born small for gestational age (SGA), BW z-scores were calculated using the Fenton growth chart for preterm infants (2013) [27]. Parents reported information about the ethnicity of the infant, maternal age, and education. Ethnicity was divided in three categories, African, Asian and mixed, as often used in similar studies [28]. Persons of African ancestry are locally considered to be Creole or Tribal. Tribal peoples are descendants of runaway slaves living in the tropical rainforest interior who recently migrated to the capital, and, therefore, tend to have less admixture with other populations than Creoles. Persons of Asian ancestry migrated from India or the island of Java to Suriname in the late 19th century and early 20th century [29]. A teenaged mother was defined as ≤19 years. Maternal educational level was considered to influence motor performance in preterm infants [30] and was divided into four categories, as low (no or primary school), low average (middle school or lower vocational training), average (high school or middle vocational training), and high (higher vocational training or university). The treating PPT reported on participation in the intervention and the number of interventions.

#### 2.2.3. Early Motor Intervention Program (EIP)

Infants were included in the EIP if they scored on the BSID-III a SS ≤ 7 for FM or GM and/or CS ≤ 80 [23]. The infants visited the PPT once a week in the first month, once every two weeks in the second month, and from there on, once every month. The same PPT (MF-P) performed all face-to-face sessions at the pediatric department of the Rehabilitation Centre of the Academic Hospital Paramaribo in Suriname.

The EIP consisted of coaching and instruction in exercise therapy. During the visits, the parents or caregivers received advice and were encouraged to perform the exercises themselves and to record the instructions and exercises on their phones. The PPT provided feedback to improve handling and exercises. The parents and caregivers were stimulated to continue the exercise therapy at home, integrated into day-to-day care. The infants were reassessed every 3 months. The EIP was stopped when a total SS for FM and GM was ≥8 or CS > 80 for two assessments in a row (see Appendix A for the topics of the intervention).

### 2.3. Statistical Analysis

Descriptive statistics were used to present cohort characteristics and motor performance outcomes of the BSID-III at *t*_0_, *t*_1_, and *t*_2_. For continuous data, we determined mean and standard deviation (SD) or median and interquartile. For categorical data, number, percentage, and range were calculated. To test for potential selection bias, an independent sample *t*-test or chi-square test was used to check for differences between the assessed and non-assessed infants at *t*_0_, *t*_1_, and *t*_2_ for gender, GA, z-score BW, AS, maternal educational level, and CS at the other time points. The same was ensured between the subgroups of infants referred and not referred for the intervention. To compare with the mean of the BSID-III reference scores of 10 for FM and GM and 100 for CS, we used a one-sample *t*-test at all timepoints.

At the three time points, data were tested for normality based on visual inspection of histogram, skewness and kurtosis for FM, GM and CS. We found no problems in skewness (−1.14 through +0.37) with all values except one between −1 and +1. Only kurtosis (+0.303 through +6.332) was too high for FM at *t*_1_, and GM and CS at *t*_2_, with the other values between −3 and +3. The normality of the residuals for the three outcome models was also checked, and both skewness and kurtosis were good. We checked for outliers and found a few at the border of −3 SD, which are reasonable values for this population and, therefore, decided to keep these in the dataset. We used mixed model linear regression analyses, which allow for analyses with missing data, without imputation or exclusion, to test longitudinal motor performance on the BSID-III for scores of FM, GM, and CS. We also determined the influence of infant and mother characteristics, perinatal and environmental factors, and intervention on longitudinal motor performance. The following factors were tested: gender, teenage mother, ethnicity of the infant, maternal educational level, GA, BW z-score corrected for GA, AS, multiple birth, ventilation, sepsis, and indication for and intervention frequency (three groups: no intervention received, 1–5 interventions, and more than 5 interventions). Selective dropout was checked by comparing characteristics of children at the different time points and whether the mean BSID-III scores differed between dropouts and non-dropouts. Both analyses showed no selective dropout. We analyzed the effect of each abovementioned factor on longitudinal motor performance with an interaction term factor * assessment moment in the mixed linear regression model. Significance of the intervention effect was tested. A value of *p* < 0.05 was considered statistically significant for all analyses, based on two-sided testing. Analyses were performed using the Statistical Package for the Social Sciences (SPSS) version 25.0.

## 3. Results

**Figure 1:**
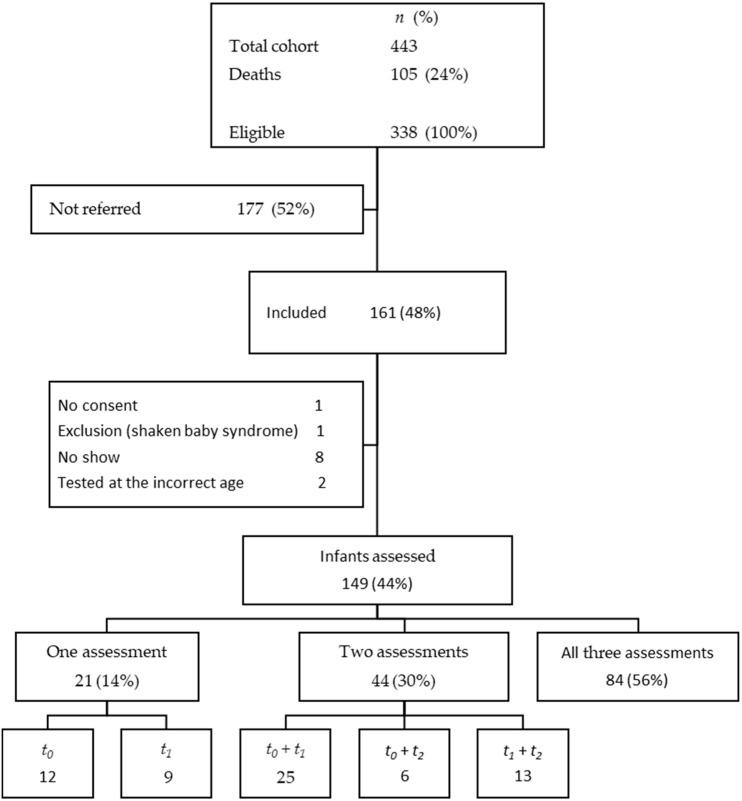
Flowchart of inclusion and assessment of referred infants at *t*_0_ = 3 months, *t*_1_ = 12 months, and *t*_2_ = 24 months.

The flowchart in Figure 1 provides an overview of the infants assessed. The inclusion criteria were met in 443 infants. Of those, 105 (24%) died before discharge. Of the surviving 338 infants, 161 (48%) were referred to the follow-up program, of whom 12 were excluded from analyses for various reasons.

Table 1 shows the characteristics of the assessed infants (*n* = 149, 49.7% boys) and their mothers and separately for the subgroups referred and not referred for the EIP. In the total group, the mean GA was 29 + 6 weeks (SD 15 days). Further, 13 infants (9%) were extremely preterm (GA < 28 weeks), 105 (70%) were very preterm (GA 28–31 + 6 weeks), and 31 (21%) were moderately preterm (GA 32–36 + 6 weeks). The mean BW was 1271 g (SD 303.9). Forty infants (26.8%) were SGA [15]. Twenty-five (16.8%) infants were a twin or triplet. The CA at t_0_ (*n* = 127) was as follows: mean (SD, range) = 3.5 months (0.77, 3–6); at t_1_, (*n* = 131) mean (SD, range) = 12.3 months (0.54, 12–15); at t_2_, (*n* = 103) mean (SD, range) = 24.1 months (0.29, 23–25).

We found no significant differences in the infants assessed or not assessed in the cohort at *t*_0_, *t*_1_, and *t*_2_ for gender, GA, z-score BW, AS, maternal educational level, and CS at the other time points. We only found significant differences (Student’s *t*-test 2.074, *p* = 0.04) for BW between the subgroups of the infants referred and not referred for the intervention. When BW corrected for GA (z-score BW) was used, no differences (Student’s *t*-test 1.727, *p* = 0.86) were found.

A total of 54 infants were referred for the EIP, of whom 12 (8.1%) did not participate and, as such, received no interventions. Therefore, 42 (78%) infants participated, of whom 28 (51.9%) received 1–5 interventions and 14 (25.9%) received 6–15 interventions. The total number of interventions differed between one and fifteen per infant. In the referred group for the EIP, the z-score of birth weight in the referred group differed significantly only at *t*_2_ as a result of the non-participation of 12 infants.

### 3.1. Longitudinal Motor Performance

**Figure 2:**
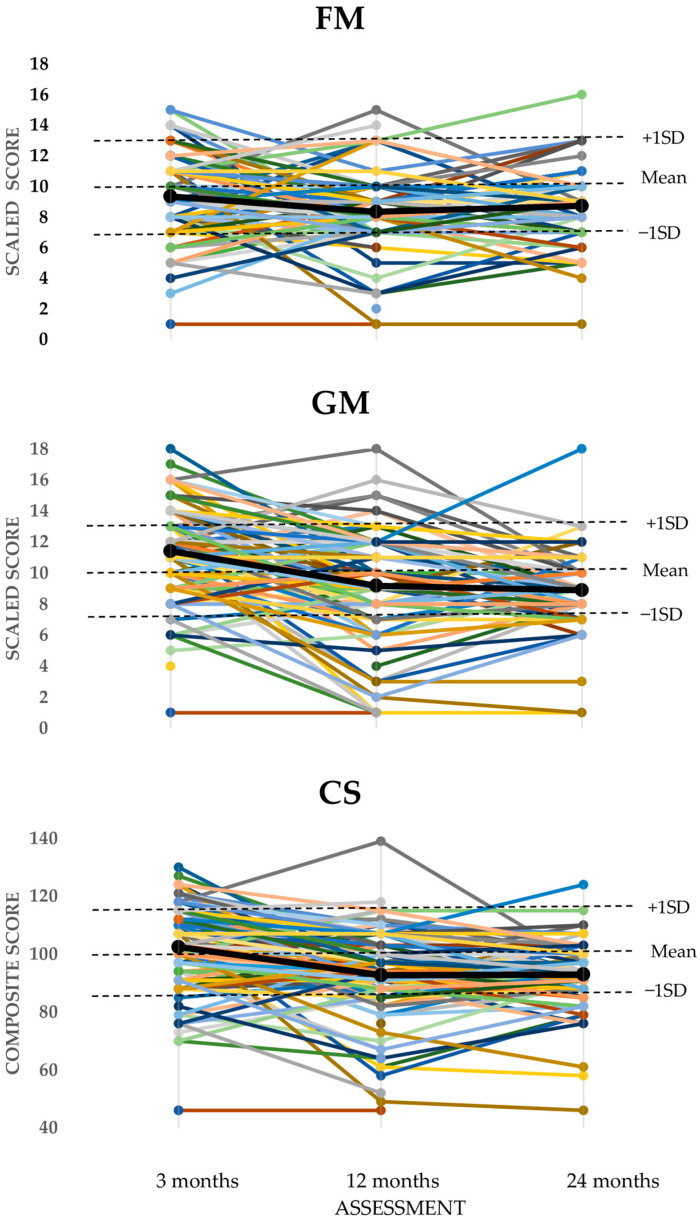
BSID-III scores for fine motor (FM), gross motor (GM) and composite score (CS) of all (*n* = 149) individual infants, with a different colored line for each individual infant; the black line represents the mean group scores, at *t*_0_ = 3 months, *t*_1_ = 12 months, and *t*_2_ = 24 months. Dotted lines represent BSID-III mean, and +1 standard deviation (SD), and −1 SD.

Figure 2 shows the individual scores of all infants (*n* = 149), with the black line representing the mean group scores. See the left part of Table 2 for the mean SSs of the BSID-III FM at *t*_0_, *t*_1_, and *t*_2_ which were 9.32, 8.36, and 8.66, respectively. The mean SSs for GM at *t*_0_, *t*_1_, and *t*_2_ were 11.39, 9.18, and 8.78, respectively. The mean CSs at *t*_0_, *t*_1_, and *t*_2_ were 102.25, 92.74, and 92.24, respectively. The mean scores of the three BSID-III outcomes decreased significantly from *t*_0_ to *t*_1_, with 0.96 points for FM, 2.20 points for GM, and 9.50 points for the CS (*p* < 0.05), and stabilized from *t*_1_ to *t*_2_. Compared to the reference values, the mean SS at *t*_0_ for FM was significantly lower (*p* < 0.01), and the mean SS score for GM was significantly higher (*p* < 0.01). The mean scores at *t*_1_ and *t*_2_ were significantly lower than the reference values for FM and GM (10) and CS (100) (*p* < 0.01).

### 3.2. Influence of Perinatal or Environmental Factors on Longitudinal Performance

Mixed model linear regression analyses were conducted to explore the influence of the selected factors. See Table 3 for the results. Of these factors, only the z-score for BW corrected for GA influenced longitudinal motor performance significantly; infants with a lower BW z-score initially had a lower score on FM and CS than infants with a higher BW z-score. However, over time, the FM and CS scores decreased less than those from the infants with a higher BW z-score, resulting in less differences at *t_2_*.

### 3.3. Influence of the Early Intervention Program

**Figure 3:**
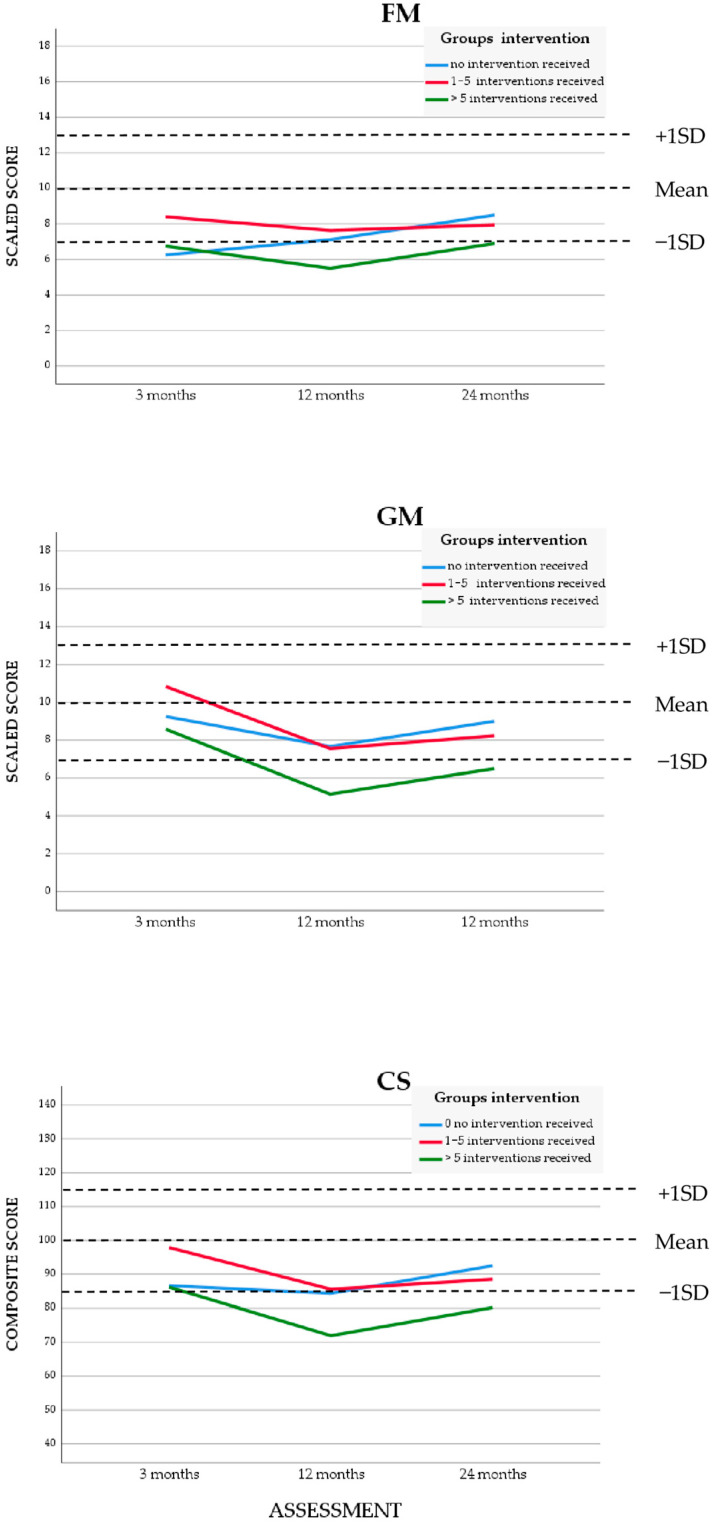
BSID-III scaled scores for fine motor (FM), gross motor (GM) and composite score (CS) of infants enrolled in the early intervention program (*n* = 54), mean group scores for frequency of intervention, at *t*_0_ = 3 months, *t*_1_ *=* 12 months, and *t*_2_ = 24 months. Dotted lines represent mean and +1 standard deviation (SD) and −1 SD of the BSID-III.

See the right part of Table 2 and Table 3 for the effect of the EIP. The mixed model linear regression analyses showed no significant effect over time for the interaction for frequency of interventions (no intervention received, 1–5 interventions, and more than 5 interventions). See Figure 3 for the longitudinal motor performance, which showed a similar course for these three subgroups. The subgroup with the highest number of interventions was also the subgroup that needed extra attention.

## 4. Discussion

This first explorative study in Suriname in VPT and/or VLBW infants showed longitudinally normal motor performance at 3 months and delayed motor performance at 12 and 24 months on the BSID-III. The BW z-score corrected for GA showed a tendency to influence longitudinal motor performance, mainly in the first year of life. Other infant and maternal characteristics and perinatal and environmental factors did not affect longitudinal motor performance. After correcting for intervention frequency, the EIP in delayed-scoring infants showed no significant interaction effects at any time point.

We controlled for bias by comparing the children’s characteristics at the different time points and the influence of score size on a missing value. We also conducted an analysis excluding the 21 children with only one measurement, reducing the drop-out from 19% to an acceptable 10%. All these analyses, with some reduced power, showed that there is no selective dropout and that the estimated effects are unbiased; therefore, we included all available data. Moreover, the missing data in the referred cohort for the yes/no EIP subgroups were also not selective. The most important limitation of this study was that 50% of the eligible infants were not referred for the follow-up program and, as such, the neonatal data of this group were missing. The bias of these missing data on the outcomes can be considerable; therefore, studies with more than 40% of missing data are generally considered only hypotheses generating. Conversely, in Suriname, almost all at-risk pregnant women give birth in one of the four hospitals in the capital, making this a representative sample for the country [17]. The drop-out rate is higher than in HICs, and this limits the generalizability of our findings to other countries.

Overall, in this study, the death rate in the neonatal period was 24%, which may also lead to biased outcomes compared to outcomes from HICs; survival of the fittest may lead to better outcomes in LMICs. Mortality rates differ by country [31]. In LICs, the age-standardized mortality rates were approximately 11.89–17.83 per 100,000 in 2019, and in HICs, the age-standardized mortality rates were between 2.02 and 2.54 [32].

The assessments of this study were started in 2014, while the Dutch version of the BSID-III (BSID-III-NL) was published in 2014, so, initially, only the US version was available. The BSID-III-NL was validated and cross-culturally adapted in Suriname in 2021, but Mc Lester-Davies did not validate the reference values [25]. Although the items in the US and Dutch version of the BSID-III motor scale are the same, the cut-off points and some starting points for age differ, with consequences for the reference values. Therefore, we chose to report the outcomes based on the BSID-III with the US reference values. Another rationale for our choice was that the ethnically diverse Surinamese population more closely resembles the US reference group [26].

The motor performance outcomes on the BSID-III CS at 12 and 24 months CA were comparable to what other studies found in VPT and/or VLBW infants, which mostly ranged from 88 to 94 [4,33]. At 24 months, CSs were similar to those reported in Vietnam [34]. At the CA of 3 months, no delays were detected for GMs and CSs. We suggest that the BSID-III CA assessment at 3 months was not sensitive enough to detect delays in this Surinamese population. There are several possible explanations for these results. First, the low number of items in the BSID-III leads to a floor effect of the test. Second, the ability to hold up the head against gravity in a prone position predominantly influences the BSID-III outcome at this age; in the current study, 35.6% infants were from African and 30.3% from Asian backgrounds, and there is limited evidence that infants from South Africa and India lift and turn their head in prone at an earlier age [35]. In addition, infants in India showed a relatively high mean BSID-III CS score (106) at 6 months, which decreased to 100 at 15 months [36]. Surinamese healthy term-born infants at 3 months also score higher on GM and CS than the US reference group [26]. We hypothesize that the method of handling the infant and demanding more head control might influence this ability.

In the neonatal follow-up, we advise the inclusion of the Prechtl General Movements assessment at 3 months to detect, at an early age, infants at risk for cerebral palsy [37,38]. Moreover, in infants with well-known risk factors for delayed motor performance and cerebral palsy such as severe chronic lung disease, periventricular leukomalacia, and intraventricular hemorrhages above grade II, or with a deviant movement quality or already diagnosed syndromes, the advice should be to start an intervention directly after discharge [18].

Clinical reasoning, including the assessment of motor performance and evaluation of movement quality, considering maternal educational level and socioeconomic factors are important factors in the decision to start an intervention also, is required in LMICs with limited resources.

### 4.1. Exploration of Influence of Perinatal or Environmental Factors

Gender, a teenage mother, the ethnicity of the infant, maternal educational level, GA, AS, multiple birth, ventilation, sepsis, and intervention did not influence the motor performance trajectories. The BW z-score (corrected for GA) showed a tendency to influence longitudinal motor performance in our study. A systematic review, including longitudinal studies mainly performed in HICs, also pointed to BW as an influencing factor on longitudinal motor performance, although, due to the limited number of suitable studies, strong evidence could not be provided [3]. Contrary to what we expected, motor performance was not influenced by GA, possibly because only 9% of the included infants were born with a GA less than 28 weeks. The mean GA of 29^+6^ weeks in our study was somewhat higher than in studies from HICs, with a mean GA of mostly 28 to 29 weeks [33]. The higher GA could have influenced motor outcomes because infants with lower gestational ages usually have more delayed motor performance and may need a longer period to catch up [10]. Twinning, a known influencing factor, was almost significant for FM performance but not for GM and CS. Our classification of the ethnic groups may have influenced the reported influence of ethnicity on motor development. In particular, differences between the subgroups with an African background and the mixed group may be too small to detect relevant differences.

### 4.2. Exploration of the Influence of an Early Intervention Program

The EIP showed no significant effect if corrected for frequency of intervention. The Cochrane review in 2024 showed some evidence that early developmental interventions improve motor development in the early years [21]. Moreover, the identification of poor motor development in the first year of life helps to identify preterm infants at risk for impairments in other developmental domains [39]. Early identification can guide early child developmental interventions, which are shown to have a favorable effect on cognitive and motor scores [22]. Therefore, we need additional studies to reach an adequate training level and adequate implementation of interventions, especially in LMICs that encounter these difficulties.

In addition, a more blended approach through video consulting or a mobile application with tutorials could be successful in improving participation and outcomes. We encouraged parents to make videos of the intervention session on their mobile phones, and that proved to be beneficial. However, parents had to come to the hospital for the intervention, and, despite a referral by the neonatologist to the intervention program, many parents did not participate. Reasons why parents did not prioritize the intervention program include (1) high travelling costs; (2) health insurance policies, in general, only reimburse ten sessions of physical therapy and occupational therapy per year; (3) parents had to take time off from work to bring the infant for treatment, consequently reducing their number of vacation days and/or income. There were only two PPTs experienced in treating young infants in the country, limiting their ability to visit the infants and their parents at home.

### 4.3. Strengths and Limitations

The longitudinal design from 3 months to 24 months with three assessment time points adds to the strength of this study. The assessments were conducted by the same experienced PPT. A limitation was that this study was not blinded but mimics daily practice in LMICs. The data collection of neonatal characteristics was mainly on paper documents. Another limitation was the lack of a control group for the intervention and potential confounding factors such nutritional status.

### 4.4. Implications for Future Studies

Future studies should include an adequate strategy that empowers parents to participate in an intervention. We advise a qualitative or a mixed method research design (combining questionnaires and interviews) to identify facilitating and limiting factors for adherence to the follow-up program and a daily exercise intervention. Moreover, neonatologists responsible for referrals to the follow-up program should be asked what is needed to improve the early referral rate. Prospective studies should preferably include infants and parents during their stay at the NICU, avoiding low referral rates. Nowadays, interviews and questionnaires via digital health techniques are available and could improve participation. A project, based on design thinking, especially taking into account the perspective of the parents, can develop the best toolbox for improving the intervention strategies in LMICs.

## 5. Conclusions

Longitudinal motor trajectories in very preterm infants (mean GA 29 + 6 weeks, SD 15 days; mean BW 1271 g, SD 303.9 g) in the MIC of Suriname showed normal motor performance at 3 months and delayed motor performance at 12 and 24 months. Mean Bayley FM and GM scores decreased between 3 and 12 months and stabilized at 24 months. Birth weight influenced longitudinal motor trajectories. Infants with a lower BW z-score initially had a lower score on FM and CS than infants with a higher BW z-score. However, over time, the FM and CS scores decreased less than those from the infants with a higher BW z-score, resulting in fewer differences at 24 months. The EIP corrected for frequency of intervention did not show influence at all time points. Clinical implementation of the motor invention program needs additional studies to reach an adequate training level. We recommend follow-up of motor performance and advise adding the Prechtl General Movement assessment at 3 months of age.

## Figures and Tables

**Table 1:** Characteristics of included infants (*n* = 149) and their mothers (*n* = 139), for no early intervention (*n* = 95) and referred for early motor intervention program subgroup (*n* = 54) and their mothers.

	Total Group (*n* = 149)		No EIP (*n* = 95)		Referred for EIP (*n* = 54)	
	Mean	SD (Range)	Mean	SD (Range)	Mean	SD (Range)
GA (weeks)	29^+6^	2.13 (25^+6^–36^+3^)	30^+1^	2.02 (25^+6^–36^+0^)	29^+5^	2.20 (26^+0^–36^+3^)
BW (grams)	1271	303.9 (640–1990)	1310	311.0 (640–1990)	1201	280.1 (730–1920) *
z-score BW *	−0.58	1.17 (−3.8–3.3)	−0.50	1.23 (−3.8–3.3)	−0.72	1.06 (−3.5–1.0)
Age mother (years)	27.2	(14–41)	27.3	(15–41)	27.1	(14–40)
	**Median**	**(Range)**	**Median**	**(Range)**	**Median**	**(Range)**
Apgar Score	9	(1–10)	9	(1–10)	8.5	(1–9)
	***n***	**(%)**	***n***	**(%)**	***n***	**(%)**
Apgar score < 7	18	(12.1)	9	(9.5)	9	(16.7)
Gender						
Boys	74	(49.7)	45	(47.4)	29	(53.7)
Girls	75	(50.3)	50	(52.6)	25	(46.3)
Z-score BW *						
SGA z-score < −1.28	40	(26.8)	29	(30.5)	14	(26.9)
AGA z-score	100	(67.2)	62	(65.3)	38	(73.1)
LGA z-score > 1.28	3	(2.0)	4	(4.2)	0	(0.0)
missing	6	(4.0)	–	–	–	–
Multiple birth						
Singleton	124	(83.2)	85	(89.5)	39	(72.2)
Twin	21	(14.1)	8	(8.4)	13	(24.1)
Triplet	4	(2.7)	2	(2.1)	2	(3.7)
Ethnicity infant						
African:	53	(35.6)	38	(40.0)	18	(33.3)
Creole	25	(16.8)				
Tribal	28	(18.8)				
Asian:	45	(30.2)	28	(29.5)	17	(31.5)
Chinese	1	(0.7)				
Hindustani	32	(21.5)				
Javanese	12	(8.1)				
Mixed and other:	38	(25.5)	21	(22.1)	17	(31.5)
Indigenous	1	(0.7)				
Mixed	37	(24.8)				
Missing	13	(8.7)	8	(8.4)	2	(3.7)
Highest maternal educational level						
Low ^a^	40	(28.8)	24	(25.3)	22	(40.7)
Low average ^b^	39	(28)	27	(28.4)	11	(20.4)
Average ^c^	36	(25.9)	27	(28.4)	13	(24.1)
High ^d^	7	(5)	5	(5.3)	2	(3.7)
Missing	17	(12.2)	12	(12.6)	6	(11.1)

Abbreviations: GA = Gestational Age; BW = Birth Weight; * z-score BW corrected for GA; SGA = Small for Gestational Age; AGA = Average for Gestational Age; LGA = Large for Gestational Age. ^a^ no or primary school; ^b^ middle school or lower vocational training; ^c^ high school or middle vocational training; ^d^ higher vocational training or university. * *p* < 0.05.

**Table 2:** Longitudinal motor performance (*n* = 149) of the fine motor (FM), gross motor (GM), and composite score (CS) and corrected for early intervention program.

	Overall Scores				Corrected for EIP ^a^	
	Mean	95% CI	Estimate of Change from *t*_0_	*p* Value	Estimate of Change from *t*_0_	*p* Value
FM				<0.001 *		0.015 *
*t*_0_	9.32	8.91–9.73				
*t*_1_	8.36	7.95–8.77	−0.96	<0.001	−0.76	0.004
*t*_2_	8.66	8.21–9.12	−0.66	0.014	−0.42	0.134
GM				<0.001 *		<0.001 *
*t*_0_	11.39	10.90–11.87				
*t*_1_	9.18	8.70–9.65	−2.20	<0.001	−1.94	<0.001
*t*_2_	8.78	8.25–9.31	−2.60	<0.001	−2.29	<0.001
CS				<0.001 *		<0.001 *
*t*_0_	102.25	99.92–104.57				
*t*_1_	92.74	90.44–95.04	−9.50	<0.001	−8.31	<0.001
*t*_2_	92.24	89.73–94.76	−10.0	<0.001	−8.55	<0.001

Abbreviations: CI = Confidence Interval; *t*_0_ = 3 months, *t*_1_ = 12 months, *t*_2_ = 24 months, EIP = early intervention program, * test of main effect of time. ^a^ After correction for frequency of interventions.

**Table 3:** Influence of perinatal and environmental factors and EIP on motor trajectories. *p* value from the mixed model linear regression analyses.

	FM *p* Value	GM *p* Value	CS *p* Value
Gender	0.442	0.164	0.179
Teenaged mother	0.630	0.292	0.304
Ethnicity infant	0.209	0.773	0.352
Maternal educational level	0.206	0.421	0.283
GA	0.497	0.394	0.304
BW (z-score)	0.013 *	0.097	0.009 *
Apgar score (dichotomized)	0.560	0.075	0.173
Multiple birth	0.065	0.935	0.300
Ventilation	0.999	0.768	0.892
Sepsis	0.402	0.095	0.102
Indication for EIP	0.293	0.718	0.340
EIP frequency	0.406	0.775	0.452

Abbreviations: EIP = early intervention program; FM = fine motor scale; GM = gross motor scale; CS = component score; GA = gestational age; BW = birth weight. * *p* < 0.05.

## Data Availability

Data are available upon request for interested researchers; requests can be directed to the corresponding author due to privacy reasons.

## Abbreviations

The following abbreviations are used in this manuscript:

VPT	Very preterm
VLBW	Very low birth weight
HIC	High income country
LMIC	Low- or middle-income country
MIC	Middle-income country
GA	Gestational age
NICU	Neonatal intensive care unit
BW	Birth weight
EIP	Early intervention program
BSID-III	Bayley scales of infant and toddler development, third version
FM	Fine motor
GM	Gross motor
CS	Composite score
PPT	Pediatric physical therapist
CA	Corrected age
SS	Scales scores
SGA	Small for gestational age
AS	Apgar score

## Appendix A. Early Intervention Program (EIP)

The intervention was provided by the same pediatric physical therapist in the country. She was educated in the Netherlands and had more than 30 years of clinical experience with children.

All sessions were face-to-face at the pediatric department of the Rehabilitation Centre of the Academic Hospital Paramaribo in Suriname.

Intervention schedule:

1. First session: explanation motor delay and recommendations for home to parents/caregivers. (±45 min)
2. First 4 weeks: once a week feedback and if necessary, adjusting recommendations. (±30 min)
3. Second and third month: once per month feedback and if necessary, adjusting recommendations. (±30 min)
4. After 3 months: repeat BSID III. (depending on age 30 to 45 min)
5. If score below average:
  - check once a month with feedback and if necessary, adjusting recommendations.
  - After 3 months: BSID III (repeat from point 4).
6. If score average or higher:
  - repeat BSID III after 3 months.
  - When BSID III on two consecutively assessments is average or higher: continue standard follow-up program.

Goal: The recommendations to parents are aimed to stimulate motor performance, and are depending on the age of the infant and the level of motor performance.

- Instructions and recommendations are given on positioning (e.g., tummy time when infant is awake), and how to elicit movements in a playful way.
- Instructions and recommendations are given verbally and are demonstrated.
- Parents/caregivers are encouraged to film the therapy sessions with their smartphone, If that was not possible it was filmed by the pediatric physical therapy department and supplied on CD/DVD.
- The parents/caregivers are then asked to practice themselves during the therapy session and coaching and feedback was given.
